# Fissurectomy with or without anoplasty for chronic anal fissures is a valid alternative to lateral internal sphincterotomy: a retrospective cohort study of 475 patients

**DOI:** 10.1007/s10151-025-03129-z

**Published:** 2025-04-21

**Authors:** G. Gallo, A. Micarelli, V. De Simone, S. Tierno, F. Tomassini, M. Goglia, A. Crucitti, M. La Torre

**Affiliations:** 1 Department of Surgery, Sapienza University of Rome, Rome, Italy; 2 Unit of Neuroscience, Rehabilitation and Sensory Organs, UNITER ONLUS, Rome, Italy; 3 Proctology and Pelvic Floor Surgery Unit, Ospedale Isola Tiberina-Gemelli Isola, 00186 Rome, Italy; 4 Department of Surgery, Ospedale Vannini, Rome, Italy; 5 Department of Surgery, Ospedale Grassi di Ostia, Rome, Italy; 6 Department of Medical and Surgical Sciences and Translational Medicine, Faculty of Medicine and Psychology, Sapienza University of Rome, Rome, Italy; 7 Department of Surgery, Ospedale Cristo Re, Rome, Italy

**Keywords:** Fissurectomy, Anoplasty, Anal fissures, Cronic anal fissures, Surgical treatment

## Abstract

**Background:**

After the failure of conservative therapy, the most effective surgical treatment for chronic anal fissures (CAFs) is lateral internal sphincterotomy. However, the choice of the procedure must be always evaluated carefully due to the risk of long-term anal continence impairment. The aim of the present study is to report the outcomes of fissurectomy with or without associated anoplasty.

**Methods:**

This study is a single-center retrospective study including patients with CAFs in whom conservative medical and non-medical treatments failed and who underwent fissurectomy with or without anoplasty from January 2015 to June 2023. Fecal continence, pain, and complications were assessed using specific questionnaires and scores.

**Results:**

Overall, 475 patients [280 males (58.9%)] with CAF underwent fissurectomy, with (*n* = 392; 82.5%) or without (*n* = 83; 17.5%) anoplasty. The majority of them (*n* = 379; 79.8%) had a posterior fissure. The mean follow-up was 49.9 months ± 26.13 months, with a minimum of 1 year, showing no intraoperative complications and a 4.2% postoperative complication rate. Missed healing occurred in 7.15% of patients at 6 months of follow-up, with higher recurrence and sphincterotomy rates in posterior fissures (*p* = 0.04). Cleveland Clinic Incontinence Scores were higher in patients who underwent anoplasty (*p* = 0.002). Interestingly, anoplasty led to a significant decrease in visual analogue scale (VAS) scores (*p* < 0.001), compared with those who did not undergo the procedure, with a consequent faster recovery.

**Conclusion:**

Our study showed that fissurectomy, whether performed with or without anoplasty, was a highly effective surgical option for treating CAFs, achieving an overall success rate of 92.8% without significant impairment of continence and with a low complication rate. Further randomized prospective trials are needed to confirm this finding. Interestingly, fissurectomy with anoplasty seems to allow for better short-term outcomes in terms of postoperative pain and recovery time compared with fissurectomy alone.

**Supplementary Information:**

The online version contains supplementary material available at 10.1007/s10151-025-03129-z.

## Introduction

The most widely accepted etiopathogenetic theory for the onset of anal fissures is the ischemic theory proposed by Schouten et al. in 1996 [1], based on the role of a high resting sphincter tone and decreased anodermal blood flow, especially at the posterior or anterior midline. For this reason, pharmacologically or surgically induced muscle relaxation is the solution most consistent with the origin of the disorder.

Most of anal fissures can be cured with a combination of conservative medical therapy and lifestyle and dietary changes [2]. In particular, calcium channel blockers (CCB) represent the most widely used drugs due to their low complication and good healing rate, as demonstrated by a recent international survey that investigated surgeons’ practices and preferences in regard to the anal fissure treatment. The survey involved 321 general and 179 colorectal surgeons, and CCB were the first-line treatment for acute anal fissures according to 59.6% of participants and for chronic anal fissures (CAFs) according to 35.8% [3, 4]. Indeed, considering the high complication rate of nitrates, which is up to 50%, representing a reason for poor therapy compliance in patients, and the non-superiority in terms of success rate, CCB still remains the first-line therapy [5, 6].

After the failure of conservative therapy, the most effective surgical treatment for CAFs is lateral internal sphincterotomy (LIS) [7]. However, the choice of this intervention must be always evaluated carefully due to the risk of long-term anal continence impairment. For this reason, a series of alternative surgical treatments, aimed at preserving continence, have been proposed.

According to the recently published guidelines on anal fissure from the Association of Coloproctology of Great Britain and Ireland, fissurectomy may be considered in association with botulinum toxin in medically resistant patients (grade of evidence: very low), whereas an anal advancement flap may be an alternative in patients with low basal sphincter pressure (grade of evidence: expert opinion) [7]. A systematic review and meta-analysis compared anal advancement flap and LIS for CAFs in 300 patients from two randomized controlled trials and two retrospective studies. The registered anal incontinence rate was higher in the LIS group [odds ratio (OR) 0.06; 95% confidence interval (CI) 0.01–0.36; *p* = 0.002], and there were no statistically significant differences regarding healing (OR 2.21; 95% CI 0.25–19.33; *p* = 0.47) and wound complication rates (OR 1.41; 95% CI 0.50–4.99; *p* = 0.51) [8].

Although we do not want to demonize LIS, in our routine clinical practice we perform the procedure only in refractory anal fissures when previous treatment fails or in patients with disease progression. For example, in the case of anal fissures complicated by abscess or fistula, a posterior sphincterotomy, despite the possibility of a keyhole deformity, might be necessary.

The aim of the present study was to show the outcomes after fissurectomy with or without anoplasty for CAFs.

## Materials and methods

### Study design

This was a monocenter retrospective study conducted between January 2015 and June 2023 in a high-volume tertiary referral center for proctological disorders. The study was developed in accordance with the Strengthening the Reporting of Observational Studies in Epidemiology (STROBE) checklist (Appendix 1) [9].

This study included 475 consecutive patients diagnosed with CAF, in whom more than 4–6 months of conservative medical and non-medical treatments had already failed [7], who presented to our outpatient clinics, and who were aged 18–75 years. Patients with acute fissures, fecal incontinence or other proctological disorders, inflammatory bowel disease, history of previous anal surgery, or a sexually transmitted disease or cancer, as well as patients who were undergoing immunosuppressive treatment, were pregnant, or were breastfeeding were excluded from the study. Patients who were unable to return for postoperative follow-up visits or showed an unwillingness to sign the informed consent form were also excluded.

In a case in which it was impossible to perform a complete proctological examination, including digital rectal examination and anoscopy, the diagnosis of CAF was performed by direct visualization during inspection of the perianal area. In cases of suspicion, a colonoscopy was performed to rule out other neoplastic or inflammatory colorectal disorders.

Apart from the temporal criterion, i.e., an onset of more than 6 weeks from the visit, the presence of at least two of the following features proposed by Scholefield et al. [10] were considered to define CAFs: a sentinel skin tag, hypertrophic anal papillae, an exposed internal anal sphincter, a fibrotic lateral fissure, or a fibrotic anal sphincter.

Patients were divided into two groups on the basis of the surgical approach: fissurectomy and diathermocoagulation with anoplasty [anoplasty group (AG)] or without anoplasty [fissurectomy group (FG)].

Fissurectomy consisted of excision of the fibrotic edges of the fissure until nonfibrotic anodermal tissue with sufficient blood support was achieved with a clear view of the internal anal sphincter. The procedure was routinely and systematically associated with the removal of both the sentinel pile/skin tag and the hypertrophied papilla. The granulation tissue at the base of the fissure was routinely scraped as a curettage and coagulated with diathermy.

Standard advancement anoplasty was performed using a flap of healthy mucosal tissue, which was mobilized and then advanced to fill the defect. The flap was secured without tension to the anal canal at 0.5–1 cm from the anal verge using three to four minterrupted 2/0 Vicryl® sutures.

In recurrent patients, the left LIS was performed with a closed method, up to the level of the apex of the fissure [11].

Patient demographics data, including age, sex, location of the fissure, and operative findings, were prospectively recorded using our database.

Postoperative complications were determined using the Clavien–Dindo classification [12]. Continence levels were assessed preoperatively, 30 days after the procedure, and 6 months after the procedure using the Cleveland Clinic Incontinence Score (CCIS), which for simplicity we call the Wexner score here [13]. Additional follow-ups were performed annually by telephone or in person if requested by the patient or if needed to exclude any possible recurrence of the disease. Pain was assessed using a visual analogue scale (VAS) (minimum score = 0; maximum score = 10) preoperatively and 7 days after the procedure.

The same surgeon (M.L.T.) performed all procedures with the patient positioned in the lithotomy position under local anesthesia with a tailored anal block [14] and mild sedation. Antibiotic prophylaxis with cephalosporine was administered to all patients. All patients underwent a minimum follow-up of 1 year.

Patients in both groups were encouraged to prevent passing hard stools and constipation by using laxatives (macrogol twice or three times a day) and a recommended oral dose of ketorolac tromethamine (10 mg every 6 h) on an as-needed basis, not exceeding 40 mg per day [6]. Moreover, patients were advised to take regular warm sitz baths, to maintain a high-fiber diet, and to increase their fluid intake up to a minimum of 2 L of water daily.

Recurrence or incomplete/missed healing was defined as the persistence of an unhealed wound after more than 6 months from the procedure. Recovery time was defined as the time (days) in which the patient was unable to fully carry out daily/work activities and used painkillers.

### Data handling and statistical analysis

The Χ^2^ test was carried out to define associations between categorical factors and groups. Given their quantitative nature, descriptive data were calculated as mean ± SD for the Wexner score, VAS, and recovery time (in days). To assess that data for independent samples, Gaussian distribution, D’Agostino *K*^2^ normality, and Levene’s homoscedasticity test were applied (where the null hypothesis is that the data are normally and homogeneously distributed). A within-subject analysis of variance was performed for the Wexner score and VAS. Sex (0 = female; 1= male), anoplasty (0 = absence; 1 = presence), and site (0 = posterior; 1 = anterior) were treated as categorical predictors, while age was treated, where possible, as continuous predictor. When subgrouping the entire sample according to anoplasty and site, a between-group analysis of variance was performed for recovery time (in days). The significant cut-off level (*α*) was set at a *p*-value of 0.05. (STATISTICA 7 package for Windows).

## Results

A total of 475 consecutive patients [280 males (58.9%); 195 female (41.1%)] with a mean age of 50.91 ± 14.95 years who were affected by CAF underwent fissurectomy with (*n* = 392; 82.5%) or without (*n* = 83; 17.5%) anoplasty between January 2015 and June 2023. The majority of them (*n* = 379; 79.8%) had a posterior site fissure (Table 1). The mean follow-up accounted for 49.9 ± 26.13 months (95% CI 47.55–52.25).

**Table 1:** Main demographic aspects and outcome measures of 475 participants

	Total patients (*n* = 475)	No anoplasty (*n* = 392)	Anoplasty (*n* = 83)	*X*^2^, *t*-test	Anterior site (*n* = 96)	Posterior site (*n* = 379)	*X*^2^, *t*-test
Age (years)	51.5 ± 14.9	48 ± 13.8	51.5 ± 15.1	*p* > 0.05	51.3 ± 14.3	50.7 ± 15.1	*p* > 0.05
Sex							
Male	280 (58.9%)	234 (59.7%)	46 (55.4%)	X^2^ = 0.13, *p* = 0.71	59 (61.4%)	221 (58.3%)	X^2^ = 0.08, *p* = 0.77
Female	195 (41.1%)	158 (40.3%)	37 (27.6%)		37 (38.6%)	158 (41.7%)	
Site							
Anterior	96 (20.2%)	79 (20.1%)	17 (20.4%)	X^2^ = 0.003, *p* = 0.95	—	—	—
Posterior	379 (79.8%)	313 (79.9%)	66 (79.6%)		—	—	
Anoplasty							
No	392 (82.5%)	—	—	—	79 (82.3%)	313 (82.6%)	X^2^ = 0.0032, *p* = 0.95
Yes	83 (17.5%)	—	—		17 (17.7%)	66 (17.4%)	
Number of complications	20 (4.2%)	17 (4.3%)	3 (3.6%)	X^2^ = 0.08, *p* = 0.77	4 (4.2%)	16 (4.2%)	X^2^ = 0.0005, *p* = 0.98
Missed healing (6 months)	34 (7.1%)	29 (7.4%)	5 (6%)	X^2^ = 0.17, *p* = 0.68	2 (2.1%)	32 (8.44%)	X^2^ = 4.18, *p* = 0.04
Sphincterotomy due to recurrence	34 (7.1%)	29 (7.4%)	5 (6%)	X^2^ = 0.17, *p* = 0.68	2 (2.1%)	32 (8.44%)	X^2^ = 4.18, *p* = 0.04

*X*^*2*^ chi-squared

No intraoperative complications occurred. The total number of postoperative complications was 20 (4.2%) (Table 2). No differences were encountered in terms of anal discharge, or pruritus between the groups. No symptoms related to the mucosal flap, such as mucosal ectropion, were registered in the AG.

**Table 2:** Frequency^*^ of complications in subgroups of patients undergoing fissurectomy with or without anoplasty

Type of complication	Group			
	No anoplasty (*n* = 392)	Anoplasty (*n* = 83)	Anterior site (*n* = 96)	Posterior site (*n* = 379)
Bleeding	6 (1.53%)	0 (0%)	1 (1.04%)	5 (1.31%)
Urinary retention	6 (1.53%)	2 (2.40%)	2 (2.08%)	6 (1.58%)
Abscess/fistula	4 (1.02%)	1 (1.20%)	1 (1.04%)	4 (1.05%)
Other	1 (0.25%)	0 (0%)	0 (0%)	1 (1.26%)

^*^Presented as *n* (%)

Missed healing was achieved in 34 (7.15%) patients at 6 months of follow-up, and LIS due to recurrence was needed in 34 (7.15%) cases (Table 1). When comparing the groups with or without anoplasty and with anterior or posterior site, no significant differences were found in terms of sex, complications, number of sphincterotomies, anoplasty, site, and age. However, subjects with a posterior fissure underwent a significantly (*X*^2^ = 4.18; *p* = 0.04) higher number of instances of missed healing and sphincterotomy (*n* = 32) compared with patients with an anterior site (*n* = 2) (Table 1).

**Fig. 1:**
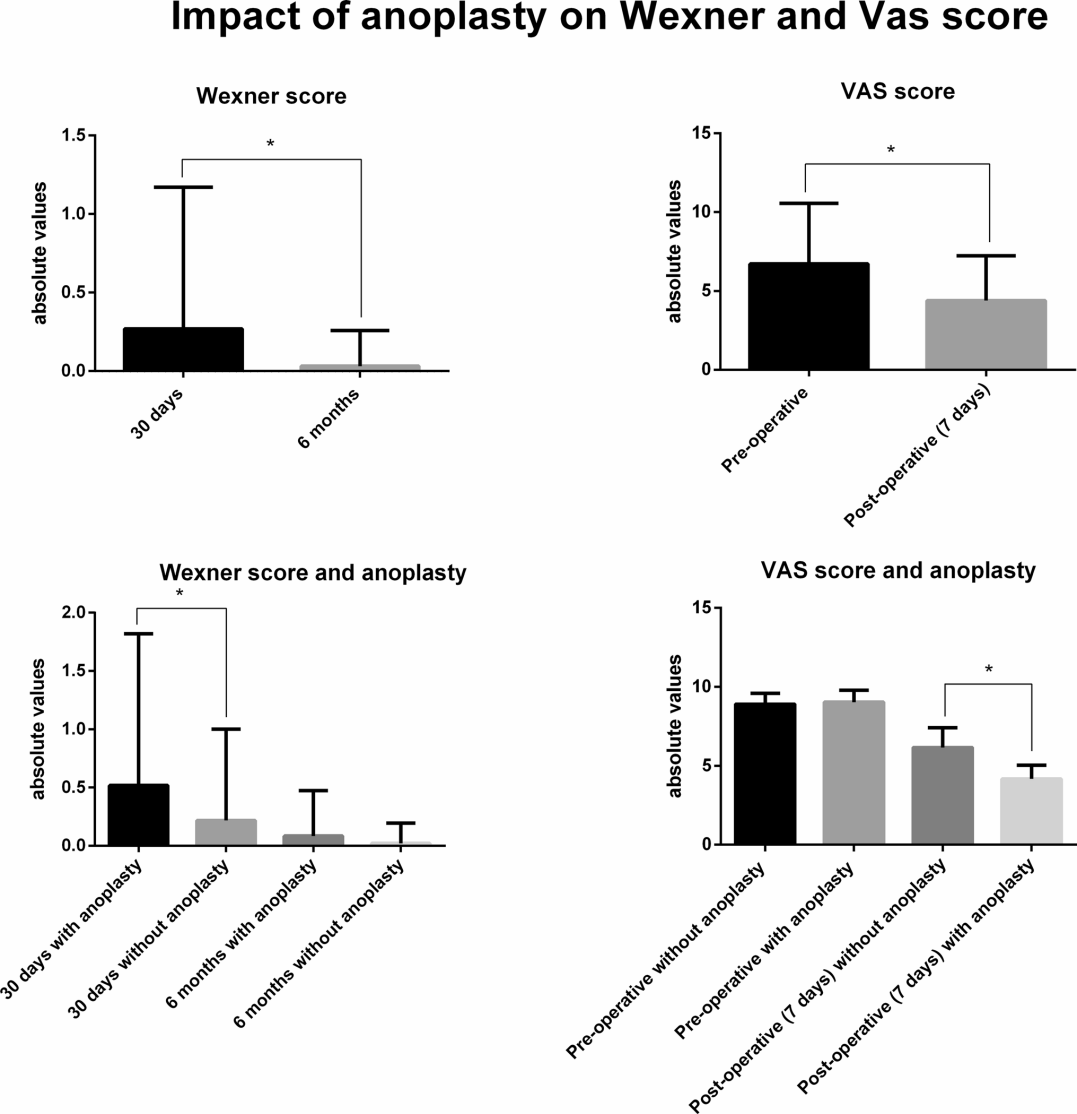
Main effects of interaction of anoplasty on Wexner score and visual analogue scale (VAS) for pain. Significant interactions (*p* < 0.05) are indicated with asterisks

The within-subjects analysis of variance demonstrated that the Wexner score significantly (*p* = 0.002) decrease by 30 days–6 months. Moreover, significant interactions were found with anoplasty, highlighting that subjects undergoing anoplasty scored worse (*p* = 0.002) on the Wexner score at 30 days compared with the others. Additionally, there was an interaction between anoplasty and sex, underlining that female patients undergoing anoplasty had a significantly (*p* = 0.011) higher Wexner score at 30 days (0.59 ± 0.08) compared with female patients who did not receive an anoplasty procedure (0.13 ± 0.61) (Table 3; Fig. 1).

**Table 3:** Within-subjects effect and interactions of Wexner score and pain assessed via the visual analogue scale (VAS) with anoplasty and sex

	Wexner score at 30 days (mean ± SD)	Wexner score at 6 months (mean ± SD)	Effect	Significance
Total patients	0.26 ± 0.9	0.03 ± 0.22		*p* = 0.002
Anoplasty	0.51 ± 1.3	0.08 ± 0.38	Anoplasty	*F*(1466) = 13.97; *p* < 0.001
No anoplasty	0.21 ± 0.78	0.02 ± 0.17		
Anterior site	0.19 ± 0.78	0.01 ± 0.1	Anoplasty × sex	*F*(1466) = 5.93; *p* = 0.015
Posterior site	0.28 ± 0.92	0.03 ± 0.24		

	Preoperative VAS score (mean ± SD)	Postoperative (7 days) VAS score (mean ± SD)	Effect	Significance
Total patients	8.92 ± 0.69	5.8 ± 1.41		*p* < 0.001
Anoplasty	9.03 ± 0.73	4.16 ± 0.88	Anoplasty	*F*(1466) = 76.55; *p* < 0.001
No anoplasty	8.91 ± 0.68	6.15 ± 1.25		
Anterior site	8.9 ± 0.69	5.9 ± 1.39		
Posterior site	8.93 ± 0.69	5.78 ± 1.42		

*SD* standard deviation

**Fig. 2:**
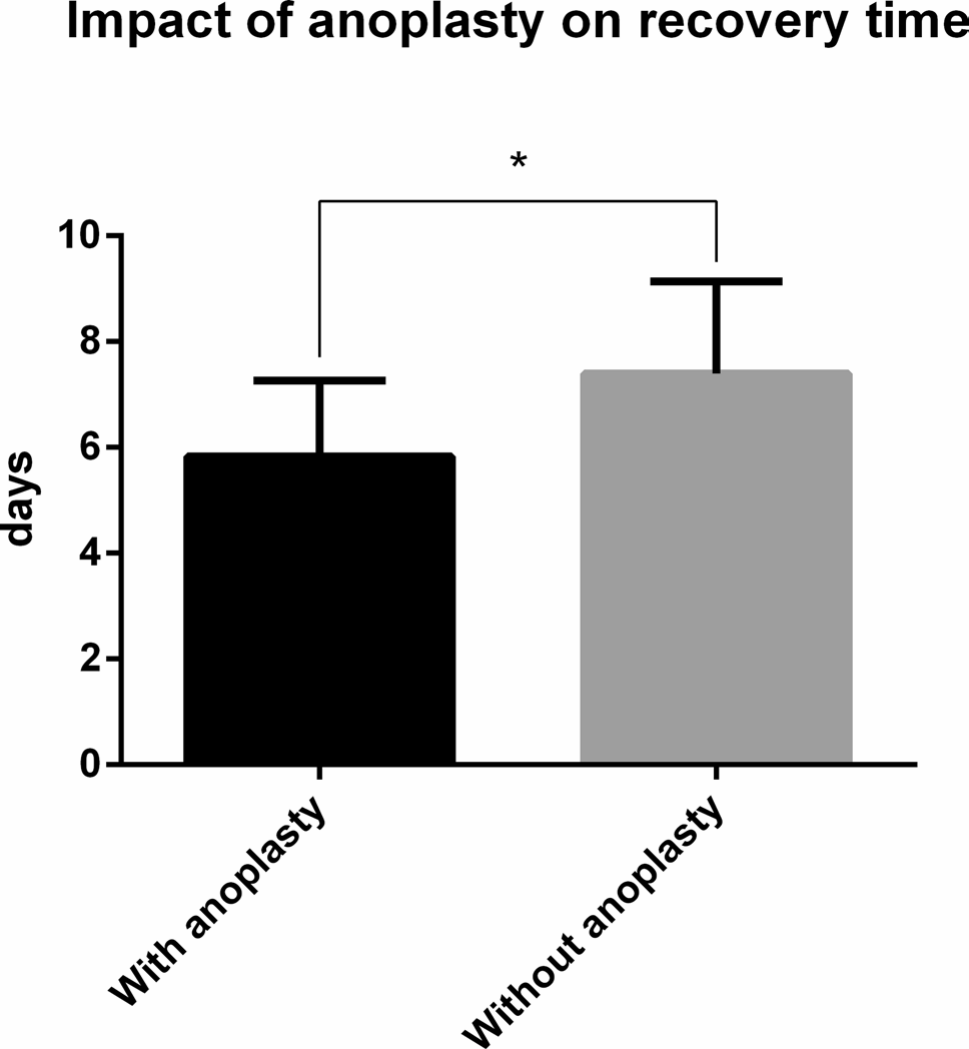
Main effect of interaction of anoplasty and recovery time. Significant interactions (*p* < 0.05) are indicated with asterisks

When considering the VAS score, a statistically significant decrease was registered (*p* < 0.001) at 7 days after the intervention compared with preoperative values. Indeed, a statistically significant interaction was also found with anoplasty, demonstrating that subjects undergoing anoplasty scored better (*p* < 0.001) on the VAS score at 7 days from the intervention compared with those who did not receive this procedure (Table 3; Fig. 1). Regarding healing time, the between-group analysis of variance found that patients undergoing anoplasty had a significantly shorter period (5.82 ± 1.42 days) of recovery compared with patients who did not undergo anoplasty (7.39 ± 1.74 days) (Fig. 2).

## Discussion

In the present study, fissurectomy with or without anoplasty was demonstrated to be a valid alternative for the surgical treatment of CAFs, with an overall success rate of 92.8% (441 out of 475). Indeed, only 34 (7.2%) patients had a missed wound healing with the subsequent need to perform a LIS. Interestingly, patients with posterior CAF had a higher incidence of missed wound healing and LIS.

As expected, anoplasty and mucosal flaps in perianal fistulas lead to minimal impairment of continence. Indeed, patients in the AG experienced a higher Wexner score than patients in the FG (*p* = 0.04). In any case, it should be considered that the preoperative Wexner score was 0, and in the postoperative period the increase, although statistically significant, did not exceed 4.

The finding of higher Wexner score values in female patients undergoing anoplasty, compared with female patients in the FG, was not surprising. In fact, the length of the internal anal sphincter (IAS) is smaller in women (2.0–3.0 cm versus 2.5–3.5 cm) [15]. However, the difference, although statistically significant probably due to the large number of patients included in the study, is not clinically significant (0.59 ± 0.08 versus 0.13 ± 0.61).

In a Dutch survey including 106 participants who were digestive surgeons and residents, the most commonly performed surgical treatment was the fissurectomy (71%), followed by LIS (27%). Interestingly, in more than half of the cases (51%), fissurectomy was combined with the injection of botulinum toxin [2].

Abramowitz et al. published a prospective, multicenter, observational study concerning the 1-year outcomes of 264 patients who underwent fissurectomy with or without anoplasty for CAF. Anoplasty was performed in 83% of the patients, and all patients achieved healing after a median time of 7.5 weeks without any recurrence at 1 year. The authors reported a de novo rate of anal incontinence of 7% after 1 year with a notable disappearance of presurgical incontinence in 15% of the patients. Of note, deterioration was associated with patients with a history of cholecystectomy, perineal tears, and an increasing number of referred vaginal deliveries. Anoplasty had no influence on healing time (*p* = 0.368) or complications (*p* = 0.154) [16]. Conversely, in our study, anoplasty reduced both healing time (*p* < 0.001) and postoperative pain 7 days after the procedure (*p* < 0.001). Possibly, the wound-lining function of the flap contributed to these effects. Interestingly, in our series, no dehiscences occurred. Moreover, in our study, no patients experienced worsening of continence at last follow-up.

Skoufou and colleagues [17] compared fissurectomy with or without anoplasty in 226 patients with idiopathic posterior anal fissure resistant to medical therapy. Both wound healing rate (93.8%) and complications rates (6.2%) were consistent with our results. In particular, there were no statistically significant differences between the two groups. For this reason, the authors concluded that anoplasty does not add any value.

Another case series by Schornagel et al. showed that fissurectomy for drug-resistant CAFs was effective in the long term (median follow-up, 8.2 years), with a low recurrence rate (11.6%) and minimal impact on continence, with a mean Vaizey score of 2.5, similar to that of the control group. The high patient satisfaction, with 90% willing to undergo the procedure again, even after a recurrence, underlines the perceived value. Preoperative continence disorders and a previous lateral sphincterotomy were associated with higher postoperative Vaizey scores, highlighting the importance of preoperative evaluation and counseling [18].

Most of the studies published in the literature so far have associated fissurectomy with or without anoplasty with botulinum toxin injection. This association lies in the pathophysiological rationale of anal fissures or rather the hypertonicity of the IAS. In fact, the toxin promotes sphincter relaxation and consequently wound healing. D’Orazio et al. [19] performed combined fissurectomy and anoplasty with V–Y cutaneous flap advancement along with 30 units of botulinum toxin injected into the IAS. Complete wound healing was achieved in all patients within 40 days from surgery. However, three local infections in the donor site and one partial dehiscence of the flap occurred. The authors also recorded the manometric finding that 5 years after the procedure was comparable to those of healthy patients.

These results were different from those of Barnes et al. [20], who reported a 66.6% complete wound healing rate (68/102) with a partial symptoms improvement in 29 patients (28.4%) after the combination of fissurectomy with the injection of 100 units of botulinum toxin. Patients in whom the procedure failed, e.g., no improvement, underwent LIS (*n* = 4) or anoplasty (*n* = 1).

This study has some limitations that need to be considered. First of all, despite the prospective enrollment of patients, the study is retrospective in nature. Second, the two groups are numerically heterogeneous but include consecutive patients of the same series reflecting real-world data. Third, this series of patients could not benefit from the use of the Scoring System for Anal Fissure (REALISE) score [21], which would have provided an objective value. However, while the score was published in 2021, the first patient of the present study was enrolled in 2016. Fourth, anorectal manometry could not be performed in all patients in the preoperative period due to pain (preoperative VAS score, 8.92 ± 0.69), and for this reason it was not included within the scope of the study. Fifth, considering the absence of alterations in the Wexner score in the preoperative period, data regarding the patients’ pregnancies were not recovered. Lastly, even if all patients met the CAFs criteria, the decision whether to perform one procedure or the other was based on the surgeon’s preference.

## Conclusions

Our study showed that fissurectomy, whether performed with or without anoplasty, was a highly effective surgical option for treating CAFs, achieving an overall success rate of 92.8% without significant impairment of continence and with a low complication rate. Interestingly, fissurectomy with anoplasty seems to allow better short–term outcomes in terms of postoperative pain and recovery time compared with fissurectomy alone. Further randomized prospective trials are needed to confirm this finding.

## Supplementary Information

Below is the link to the electronic supplementary material.

Supplementary file1 (PDF 113 KB)

## Data Availability

No datasets were generated or analyzed during the current study.
